# Regression to the Primitive Stage: A Case Report

**DOI:** 10.7759/cureus.88525

**Published:** 2025-07-22

**Authors:** Ezequiel Lafont, Lubov Leontieva

**Affiliations:** 1 Psychiatry, Icahn School of Medicine at Mount Sinai, New York City, USA; 2 Psychiatry, State University of New York Upstate Medical University, Syracuse, USA

**Keywords:** anti-psychotics, psychological defense mechanism, psychosis, regression, schizophrenia and other psychotic disorders

## Abstract

We describe a unique case of a previously independent young male with acute psychosis who experiences various stressors resulting in severe functional decline, demonstrated by fecal and urinary incontinence, impaired ability, and disregard for articulate needs, resembling the early developmental stage of infancy. The patient’s regression symptoms were responsive to antipsychotics, suggesting regression may represent an under-recognized symptom of acute psychosis, though this remains speculative until other studies further explore this phenomenon.

## Introduction

Regression is a well-known psychological defense mechanism introduced by Sigmund Freud in one of his prominent works, The Interpretation of Dreams [1]. It is characterized by an unconscious return of the ego to a previous stage of development and is seen as a coping strategy explained by immature behavior in response to periods of stress [2]. In toddlers, regressive behaviors can be seen when a child is overwhelmed or reaches or passes a new milestone; though these moments are often temporary and can resolve in several weeks by identifying the underlying cause [3]. Regression in adults also occurs and can result from varying environmental stressors or neurological conditions such as head trauma and focal brain lesions in the frontal lobe; however, evidence to support psychosis-induced regression is limited [4-6]. While clinical manifestations between normal developmental regression and adult regression are similar, the difference lies in the life stage at which it is expressed. In toddlers, regressive behaviors are typical and expected, while in adults, these behaviors tend to be pathological [4]. Its phenomenological profile takes on various features ranging from cognitive to emotional and behavioral forms [4]. From a behavioral perspective, regression commonly manifests as childlike behavior, exhibited by temper tantrums, baby talk, incontinence, and thumb sucking, amongst other displays [4]. Emotional features of regression can be displayed as increased vulnerability or emotional dysregulation, such as rapid mood swings. Cognitive features can be displayed as disorganized reasoning and magical thinking to provide a sense of security for oneself in moments of vulnerability. All of the manifestations described are represented in early childhood stages and mimic regressive-like behaviors. 

Psychodynamic mechanisms of regression ascribe to a process where the ego becomes overwhelmed by either internal or external stressors, loses contact with reality, and, as a result, retreats to a primitive state where the ego may feel more protected and less vulnerable [1]. While the phenomenon of psychological regression is rooted in psychoanalytic theory, contemporary neurobiological frameworks offer different perspectives. Developmental trauma frameworks describe how regression can be exhibited as a result of neurodevelopmental traumas, where the brain reverts to primal neural circuits under stress, hindering higher-functioning circuits from reacting [7]. Neuropsychiatric frameworks discuss regression in connection with disruptions in frontal lobe processes and higher executive functioning that impair insight and organized thinking, which can present as symptoms of regression [8,9]. The traumatogenic phenotype of psychosis is a term used to describe individuals who experience developmental traumas and exhibit quantitative and qualitative psychotic symptoms different from those who have not experienced developmental trauma [10]. Through systematic review, a pattern demonstrating those with developmental traumas marked by sexual, emotional, and physical abuse and neglect in a household under 18 years of age appeared with more severe positive, depressive, and anxious symptoms than those without developmental trauma and were at greater risk of treatment dropout and hospital readmission as psychosis worsened over time [10]. 

In searching the literature, regression to a primitive stage in acute psychosis is scarce, perhaps due to both the nuance and absence of the symptom as a formal diagnostic criterion for psychotic disorders [11]. One case report describes a patient with schizophrenia who, after multiple overwhelming traumatic stressors, sought psychiatric and psychoanalytic treatment for suicidal ideation [12]. The patient entered a six-month state of psychotic regression characterized by hypochondriasis and delusions of blindness, believing he was unable to see, though psychologically, his perceived loss of sight was one stemming from an inability to work through his complex traumatic relationships, the most notable one with his mother [12]. His psychotic regression was heavily treated and improved with intensive psychoanalytic psychotherapy and limited pharmacotherapy, as the patient disliked the medication’s side effects. Therapeutic interpretations allowed him to have greater insight into his paranoia and delusions and re-establish his ego capacities [12]. Another case report details a patient admitted for acute psychosis following a recent (one year prior) post-traumatic accidental mutilation of his fingers from a work-related injury [13]. His psychosis was characterized by auditory and visual hallucinations, irritability, and difficulty performing activities of daily living [13]. The patient was prescribed ziprasidone 40mg twice daily oral and lorazepam 1mg twice daily oral, with ziprasidone later switched to amisulpride 400mg twice daily oral, given visual disturbances persisted with the initial treatment. His psychotic symptoms resolved on this regimen, along with cognitive-oriented psychotherapy post-hospitalization, though post-traumatic stress disorder (PTSD) symptoms persisted and highlight an overlap between PTSD and psychotic features [13]. 

Primitive regression is primarily an observed phenomenon, making it difficult to quantify and measure against classic psychotic features, though the characteristics of regression and the neurobiological mechanisms involved in neurodevelopmental traumas suggest a link between regression and symptoms of psychosis. Lack of psychoanalytic presence in contemporary psychiatry may also play a role in detecting this particular feature in acute psychosis, as psychological regression is rooted in psychoanalytic theory, and the majority of psychiatry residency training programs have minimal exposure in this subspecialty [14]. 

Below, we describe a case report of a young male diagnosed with unspecified schizophrenia spectrum disorder who was admitted for acute psychosis. The patient, previously independent, experiences various stressors resulting in severe functional decline demonstrated by fecal and urinary incontinence with impaired ability and disregard to articulate needs, mirroring the primitive years of an infant. The main objective of this case was to illustrate the antipsychotic treatment response to primitive regression and how this phenomenon may be an under-recognized symptom of psychosis.

## Case presentation

Our case describes a young male in his late twenties with a past psychiatric history of PTSD, schizophrenia versus schizoaffective disorder, and a history of sexual abuse by family members and friends between the ages of five and eight. He reports a maternal psychiatric history of schizophrenia versus schizoaffective disorder and expresses a strained relationship with his mother, marked by episodes of physical abuse; his father remains absent. The patient’s initial encounter with a psychiatrist occurred during the sixth grade, when he was formally diagnosed with attention deficit hyperactivity disorder and depression. He was lost to follow-up, and his child and adolescent history remains unclear, though in July 2016, he sustained a head injury requiring a craniotomy for subdural hematoma evacuation. 

Up until his recent and subsequent hospital encounters, the patient was able to perform activities of daily living and function independently. He successfully completed three years of college and was involved in extracurricular activities, playing multiple musical instruments and performing as a drummer in a heavy metal band. He also maintained employment in the supermarket and restaurant industries. However, as records indicate, from June 2024 to February 2025, he presented to the emergency department twenty-six times with varying complaints. The patient’s first four visits, all spanning one month, presented as suicidal ideation secondary to homelessness. Eviction from his partner’s home subsequently led to his undomiciled status, resulting in severe functional decline regarded as atypical for an individual who was otherwise fully independent. He became incapable of taking care of his basic needs, frequently incontinent of urine and feces, and unable to remember how to navigate the transit system. He was banned from all shelters and warming centers in the region due to his heavy incontinence and poor hygiene. Case workers were concerned about his risk of imminent death from hypothermia. In February 2025, the patient was brought in by mental health services for concerns of safety in the community and was ultimately admitted for psychiatric stabilization secondary to posing an imminent risk to self. 

During his admission, the patient exhibited poor hygiene, demonstrated by an ungroomed appearance, malodor, and dry, flaky skin. He was withdrawn, had minimal verbal output during interviews, and intermittently responded to internal stimuli. He was incontinent of urine and feces and would remain soiled until cleaned by staff. The patient was selectively mobilized for food but would stay in bed for the majority of his hospitalization. His mental status examination, though limited due to his volitional unresponsiveness and uncooperativeness during interviews, was consistent throughout his stay, creating challenges with assessment. The patient was awake, alert, and appeared older than stated age, with flaky skin, matted hair, and poor grooming. He exhibited minimal motor movement and poor eye contact. Speech was normal in quality, articulation, and volume, and when asked about his mood, the patient would decline to respond, and affect remained flat. Thought process, insight, and judgment were difficult to assess based on limited interactions, and he would appear intermittently preoccupied. He was given olanzapine 10 mg orally at night. If he refused this, given that the patient was granted Treatment Over Objection (the administration of medical treatment when a patient is incapable of consenting), he then received 10 mg intramuscularly and had notable improvement in his condition in four days, becoming more animated, cooperative, engaged, and active. During this time, he started utilizing the bathroom to urinate and defecate, taking occasional showers, and inquiring about changing his bed linen. After medication was enforced, the patient started sitting in a chair and showed markedly improved facial and skin hygiene. He continued to be awake and alert and would engage in conversation, with thought processes being grossly linear. He did not endorse suicidal ideation or homicidal ideation, though insight and judgment were poor. The patient stopped appearing internally preoccupied and did not endorse auditory or visual hallucinations. When asked about his mood, the patient would frequently state, “I want to get out of here.” Affect was dysphoric, irritable, and flat. Given his improved clinical status, the medical team determined his treatment goals were met, and the patient was deemed stable for discharge (Table 1).

**Table 1:** Timeline of the patient’s condition and outcome

Age	Event	Outcome
Ages 5-8	Experienced sexual abuse by family members and friends	No intervention
Ages 11-12	First encounter with a psychiatrist and diagnosed with attention deficit hyperactivity disorder (ADHD)	Lost to follow-up
~Age 19 (July 2016)	Sustained a head injury requiring a craniotomy for subdural hematoma evacuation	Surgical intervention
~Ages 18-22	Attended three years of college, participated in extracurricular activities, and held employment in the grocery/restaurant industries	No intervention
~Ages 27-28	Eviction from his partner’s home led to an undomiciled status	Severe functional decline
Age 28 (June-July 2024)	Presented to the ED four times with complaints of suicidal ideation as a result of eviction	Psychiatric stabilization, discharged
Age 28 (June 2024-Feb 2025)	A total of twenty-six ED visits for varying complaints as a result of eviction	Psychiatric stabilization, discharged
Age 28 (Feb-March 2025)	Admitted to the mental health services to the inpatient psychiatric unit for psychiatric stabilization, secondary to safety in the community	Prescribed Zyprexa 10mg PO with poor adherence
Age 28 (February 2025)	Treatment Over Objection granted	Significant improvement in hygiene, social engagement
Age 28 (March 2025)	Treatment goals met	Stable for discharge

## Discussion

Humans are extraordinarily vulnerable beings who are at risk of being psychologically damaged by shortcomings and, in response, develop psychological defense mechanisms to circumvent difficult situations. These mechanisms serve as protective strategies and the mind’s way to adapt to hostile environments [15]. In our case described above, the patient experienced a surge of anxiety to which he regressed to the primitive stage. Antipsychotics aided in reintegrating his ego back together in order to break out of his regression state. From a psychodynamic perspective, the fragmented ego was “glued together” with medication and played a role in stabilizing the patient’s psychosis [16]. From a neurobiological lens, antipsychotics’ pharmacodynamic properties helped mediate dysregulated neural circuits in order to restore the patient’s baseline level of functioning. 

The pharmacodynamics of olanzapine help in understanding the improvement in patient symptoms. Olanzapine is a second-generation antipsychotic used to treat schizophrenic persons over the age of thirteen and acute manic and mixed episodes in bipolar I disorder [17]. It acts on dopamine D2 and serotonin 5HT2A receptors in the brain and can treat both positive and negative symptoms seen in schizophrenia. In the mesolimbic pathway, dopamine is released and activates protein kinase A, which is responsible for excitatory responses resulting in positive symptoms. Olanzapine’s D2 antagonistic effects also inhibit this pathway to cause a decrease in positive symptoms that can present as hallucinations and delusions. Similarly, serotonin 5HT2A binds to 5HT2A receptors to produce excitation in the prefrontal cortex. The prefrontal cortex is an area involved in executive functioning, emotion regulation, and cognition, and olanzapine’s disinhibition of dopamine release results in a decrease of negative symptoms, including flat affect, anhedonia, and poor attention [18]. In our case described, the patient’s disorganization and behavioral regression from acute psychosis were stabilized with antipsychotics through their multi-receptor binding activity. The regression seen in this case, exhibited by urinary incontinence, minimal responsiveness, selective mobilization, and dishevelment, are behaviors that involve regions of the frontal cortex and are implicated in the pharmacodynamics of olanzapine. The therapeutic properties of olanzapine helped restore the patient’s higher order of executive functioning, and while the term regression does not strictly fall into the positive or negative diagnostic criterion of psychosis, his presentation and responsiveness to antipsychotic treatment suggest an association between regression and psychosis. 

Our case demonstrates the use of an antipsychotic to treat symptoms of regression in the context of acute psychosis and can be explained through various frameworks. The patient experienced an overwhelming stressor leading to incontinence and inability to communicate his basic needs, mirroring the behavior of an infant and entering a state of “learned helplessness,” a psychological condition in which an individual is resigned to any control of one’s circumstances after repeated exposure to a stressor [19]. The repeated exposure to chronic stress and, ultimately, the overwhelming trigger of homelessness may have resulted in a default reaction of passive behavior and anxiety, a redefined approach to learned helplessness [20]. This modern framework, which is neurobiologically supported in depression and PTSD patients, takes the perspective that helplessness is not learned but rather a neurological response to repeated uncontrollable events [20]. Given the patient’s psychotic personality type, the utilization of immature defense mechanisms is to be expected [21]. His primitive regression also brings about discussion on distinguishing trauma-based dissociation from freeze response in the lens of acute psychosis, as the two may present with similar symptoms but are rooted in different mechanisms (physiological vs. psychological). An overwhelming traumatic experience can cause trauma-based dissociations where the mind detaches into disconnected parts in order to escape the distress and can appear as acute psychosis [22]. By contrast, a freeze response due to a traumatic event can result in the sudden halt of the parasympathetic nervous system, causing the body to freeze in the moment and induce an autonomic reaction such as a halt in movement and speech [23]. 

The differential diagnoses for regression are wide-ranging and include poor coping, catatonia, psychotic disorders, dissociative disorders, major depressive disorder, and delirium, to name a few [4]. Psychosis-associated incontinence, though uncommon, can occur in acute psychosis and catatonia [24,25]. While we mainly attribute his primitive regression to psychosis, given his homelessness and subsequent severe functional decline, we recognize that the etiology for his presentation is likely multifactorial. His continued exposure to psychological trauma as a child, his traumatic brain injury (TBI) as a teenager, and his ultimate undomiciled status resulting in severe functional decline all serve as confounders that complicate diagnosis. Complex PTSD secondary to child sexual assault and persistent sexual abuse suggests a prolonged period of trauma that could result in problems with behavioral regulation [26]. TBI may have also contributed to his decline in functioning, given the variable latency of presentation [27], and short- and long-term effects of TBI can affect reasoning, language, and general intelligence [28]. 

He was ultimately diagnosed with unspecified schizophrenia spectrum disorder as a diagnosis of exclusion, given his presentation did not meet the full criteria for categories within schizophrenia spectrum and other psychotic disorders [29]. While catatonia was considered a differential diagnosis, elements of the clinical presentation were inconsistent with this diagnosis. The patient scored low on the Bush-Francis scale, was reactive to environmental stimuli, and exhibited volitional movement [30].

Strengths and limitations

One strength of this case report is patient treatment responsiveness to an otherwise limited studied presentation. In contrast, a notable limitation includes the patient being a poor historian with an unclear psychiatric timeline. Despite a psychiatric history being unclear, his severe functional decline from baseline is evident. The patient’s behavioral regression was improved with antipsychotics; however, further studies are warranted to explore the role of antipsychotic medication in treating regression as a symptom of psychosis.

## Conclusions

This case highlights a unique presentation of psychosis through regression to infantile behavior and may be associated with reversibility of symptoms with antipsychotic treatment. It illustrates the multifactorial nature of primitive regression in acute psychosis and how various neurobiological, trauma-informed, developmental, and psychological frameworks are implicated in symptom treatment. While this phenomenon was observed in this case, correlation does not imply causation, and this association remains speculative, which highlights the need to further explore this topic. Prospective cohort studies, neuroimaging, and case series would be beneficial in strengthening associations.
